# Haves and have nots must find a better way: The case for open scientific hardware

**DOI:** 10.1371/journal.pbio.3000014

**Published:** 2018-09-27

**Authors:** André Maia Chagas

**Affiliations:** 1 Werner Reichardt Centre for Integrative Neurosciences, University of Tübingen, Tübingen, Germany; 2 Graduate School for Neural and Behavioural Sciences, University of Tübingen, Tübingen, Germany; 3 TReND in Africa gUG, Bonn, Germany; 4 Institute of Ophthalmic Research, University of Tübingen, Tübingen, Germany; 5 School of Life Sciences, University of Sussex, Brighton, United Kingdom

## Abstract

Many efforts are making science more open and accessible; they are mostly concentrated on issues that appear before and after experiments are performed: open access journals, open databases, and many other tools to increase reproducibility of science and access to information. However, these initiatives do not promote access to scientific equipment necessary for experiments. Mostly due to monetary constraints, equipment availability has always been uneven around the globe, affecting predominantly low-income countries and institutions. Here, a case is made for the use of free open source hardware in research and education, including countries and institutions where funds were never the biggest problem.

## Abstract 2

In 2013 Eve Marder raised concerns about research equipment availability and the divide between well-funded and poorly-funded labs. Five years later, with the issue still present, this Perspective article discusses a possible solution: the wide adoption of Open Source Hardware, not only to reduce the divide, but also to make research and education more robust, reliable and widespread.

In 2013, Eve Marder [1] expressed concerns about the increasing costs of equipment necessary to do state-of-the-art research in the field of biology and the decreasing amount of funding available to be shared between an ever-growing number of labs and researchers. Even though the funding situation in the United States has improved since 2013, a closer look shows that the investments accumulated an inflation of 9% in the period between 2012 and 2017 [2] and a budget increase of 4.5% and 6% for the National Institutes of Heath and National Science Foundation, respectively [3] [4], while the Environmental Protection Agency has had a cut of 4.5% in the same period [5]. The concerns raised by this situation have been expressed for quite some time in many places of the world, where lower investment (as a proportion of gross domestic product [GDP]) in science and education makes research conditions suboptimal and access to bleeding edge technology and tools difficult. Now, in times of shrinking funding, however, this difficulty is being felt by researchers in places earlier considered safe havens of science. One of the fears Professor Marder expressed is that we might return to the “old days,” when only a privileged few men were able to do research.

A major reason for high prices in scientific equipment is related to the way innovation, technological development, and new knowledge generated inside universities and research institutes are introduced to the world: to make them commercially interesting and to allow their development outside academia, they are protected using legal mechanisms such as patents and/or copyrights, which are then licensed/sold to companies. Although this system enables universities and companies to work in collaboration and leverage each others’ strengths, it ends up locking away research results funded with public money. Not only is this morally debatable, but it also does the following:

1. increases costs in product development, as each patent normally requires the involvement of specialised lawyers and has several fees that can easily sum up to considerable amounts (between US$10,000 and US$30,000 in 2018 [6]);
2. slows down innovation cycles, as sometimes companies owning patents do not invest in further developing them into commercial applications, delaying derivative innovations that would rise from its implementation [7]. Kodak for example patented [8] the idea for a charge-coupled device (CCD) digital camera (the same as today’s digital cameras) in 1978, but the first commercial versions were only released by Fuji in 1989 [9];
3. creates instruments/technologies that are “black boxes” since consumers are not allowed to open them up (for repair, maintenance, or simple curiosity), which, in the case of scientific equipment, can lead to an incomplete understanding of how complex instruments work as well as their capabilities and limitations;
4. and equipment ends up being used in suboptimal conditions or thrown away instead of being repaired, especially in countries where manufacturing companies do not offer appropriate customer support [10].

An alternative to this production model exists, and it relies on a free distribution philosophy, in which code and design blueprints are shared freely so that anyone can study, build, modify, and improve existing projects. This philosophy is strongly present in the software industry, more known as open source, in which several companies act as service providers for their products instead of leveraging scarcity and intellectual property [11]. One example of such a company is Red Hat, which was founded in 1993 and has, as of 2015, reported over 2 billion dollars in revenue yearly for the last three years [12].

As a consequence, researchers can now use freely available software for most of their work-related tasks (e.g., office suites, statistics, or data analysis packages), which in turn helps reduce research costs and frees resources for other expenses (as well as improves research quality and scientific outputs [13]—a more detailed description of the benefits of using freely available initiatives can be found in the following paragraphs). For Eve Marder’s main concern—the lack of funds to buy expensive equipment—a solution was still lacking. While this is worrisome, the increasing affordability of electronic systems to the general public should provide relief to the problem, as they can be used to assemble tools in an open source way in which everyone is free to use and improve designs based on their specific needs. Smartphones, for example, carry powerful central processing units (CPUs), camera, microphone, and an array of sensors, which makes these ever-present devices excellent tools for recording, analysing, and visualizing data [14]. One popular application is smartphone-based microscopes, in which a glass bead or other inexpensive plastic lens (normally found inside a laser pointer) can be placed in front of the smartphone camera for very large magnifications (see Table 1 for examples). A brief online search for “phone microscope” will guide the reader to a wealth of similar projects. The quality of such simple microscopes allows users, among other things, to image blood samples for diagnostics [15], with costs in the range of US$5–US$20 (assuming users already have a smartphone with a camera).

**Table 1:** 

Smartphone-based microscopes	
PNNL Smartphone Microscope	https://availabletechnologies.pnnl.gov/technology.asp?id=393
US$10 smartphone-to-microscope conversion	http://www.instructables.com/id/10-Smartphone-to-digital-microscope-conversion/
PhoneScope	https://www.thingiverse.com/thing:280004
LudusScope	http://journals.plos.org/plosone/article?id=10.1371/journal.pone.0162602
Smartphone clip-on microscope	https://www.amazon.com/KINGMAS-Microscope-Magnifier-Universal-Smartphones/dp/B00PQ9XV2E
Open source optical microscopes	
FlyPi	http://journals.plos.org/plosbiology/article/metrics?id=10.1371/journal.pbio.2002702#citedHeade
Open Source Multifluorescence System	http://journals.plos.org/plosone/article?id=10.1371/journal.pone.0187163
OpenFlexure Microscope	http://tutorial.waterscope.org/
Foldscope	https://www.foldscope.com/
The Open Source Microscope	http://openlabtools.eng.cam.ac.uk/Instruments/Microscope/
Public Lab Basic Microscope	https://publiclab.org/wiki/basic-microscope
Free and open source automated 3D microscope	http://onlinelibrary.wiley.com/doi/10.1111/jmi.12433/full
Hackaday.io list on optical microscopes	https://hackaday.io/list/12057-optical-microscope-projects
Organizations using FOSH for education	
TReND in Africa	http://trendinafrica.org/
Biohackacademy	http://biohackacademy.github.io/bha2/
Lego2Nano	http://lego2nano.openwisdomlab.net/index.html
Public Lab	https://publiclab.org/
PhotosynQ	https://photosynq.org/education
Conector Ciência	http://www.conecien.com/
ScienceXplore	https://www.sciencexplore.org/
Backyard brains	http://blog.backyardbrains.com
FOSH for science (publications, companies, and nonacademics)	
Smart tube holder for centrifuges	http://journals.plos.org/plosone/article?id=10.1371/journal.pone.0195907
Optics	http://journals.plos.org/plosone/article?id=10.1371/journal.pone.0059840
Defibrillator	https://www.sciencedirect.com/science/article/pii/S2468067217300354
Microfluidic thermometer	http://journals.plos.org/plosone/article?id=10.1371/journal.pone.0189430
Time sorting pitfall trap	https://www.sciencedirect.com/science/article/pii/S2468067216300220?via%3Dihub
Automated four-point probe	http://www.mdpi.com/1996-1944/10/2/110
Pico Spritzer	https://www.nature.com/articles/s41598-017-02301-2
pH Stat	http://journals.plos.org/plosone/article?id=10.1371/journal.pone.0193744
Ultrasonic signal generator	https://arxiv.org/abs/1610.00492
Automated feeder	https://www.sciencedirect.com/science/article/pii/S2468067216300050?via%3Dihub
Plant tissue culture system	https://plantmethods.biomedcentral.com/articles/10.1186/s13007-017-0156-8
PCR machine	http://openpcr.org/
US$5 PCR machine	https://hackaday.io/project/1864-5-dna-replicator
Electrophysiology system	http://www.open-ephys.org/
Raspberry Pi-based supercomputer I	http://www.southampton.ac.uk/mediacentre/features/raspberry_pi_supercomputer.shtml
Test Tube photometer	http://openplant.science/2017/12/09/photometer-shopping-list.html
Generic lab equipment	http://www.gaudi.ch/GaudiLabs/?page_id=328
Scanning electron microscope	http://benkrasnow.blogspot.de/2011/04/diy-scanning-electron-microscope-image.html
Prostheses I	http://www.openbionics.org/
Prostheses II	http://enablingthefuture.org/
Water quality-testing platform	http://www.appropedia.org/Open-source_mobile_water_quality_testing_platform
FOSH repositories	
Appropedia	http://www.appropedia.org/
Hackaday	www.hackaday.io
Hackteria	http://hackteria.org/
BioHackacademy	https://github.com/BioHackAcademy
PLOS Channels–Open Source Toolkit	https://channels.plos.org/open-source-toolkit
Openeuroscience	www.openeuroscience.com
Github	https://github.com
Gitlab	https://gitlab.com/
Open Plant Science	http://openplant.science/
Journal of Open Hardware	http://openhardware.metajnl.com/
HardwareX	https://www.journals.elsevier.com/hardwarex
KitSpace	https://kitspace.org/
DocuBricks	https://www.docubricks.com/
Wevolver	https://www.wevolver.com/discover
Open Hardware Repository	https://www.ohwr.org/projects

**Abbreviations:** FOSH, free open source hardware; PNNL, Pacific Northwest National Laboratory.

In contrast to this, the initial cost for a “scientific-grade” optical microscope is in the range of thousands of dollars, with prices rising steeply for more complex designs, severely constraining access to such a fundamental tool. As such devices are core to scientific investigations, there have been several open source models beyond just the “basic smartphone hack;” some examples can be found in Table 1. They have different capabilities and different levels of complexity, but all of these freely distributed models have two key features in common: (i) they are produced with “off-the-shelf” components, which are mostly cheap and easy to get, and (ii) their designs and bill of materials are available online, allowing anyone to build as well as customize/improve them, depending on specific needs and material availability [16]. These features are nothing more than the translation of the open source software philosophy to the world of hardware (a more rigorous definition can be found on the Open Source Hardware Association page [17]). Like in software, the adoption of this philosophy in research and education has deeper implications, which have been debated in reference to specific fields (analytical chemistry [18], engineering [19], life sciences [20], and nanotechnology [21]) as well as concerning research in general [22,23]. The common implications of adopting free open source hardware (FOSH) are summarized below:

1. It allows more people to participate in the scientific endeavour, in turn enabling research to be done outside academia, enabling people to exercise their curiosity and better understand the world around them. Public Lab [24] and Safecast [25] are two good examples of nonprofit organizations that use FOSH to empower global communities to gather data about environmental variables and to understand the impact of human activity on the environment, health, and quality of life.
2. It allows for a better understanding of the tools themselves because the available blueprints can be studied, leading to more informed decisions by users concerning the feasibility of experiments and the results they can expect. This, in turn, can also lead to better reproducibility, as researchers can calibrate their devices according to the blueprints more often and make sure they are performing consistently at high standards, avoiding discrepancies in experimental outcomes.
3. In long-term projects lasting years or even decades, laboratories are less vulnerable to supply problems. If a company producing a certain device decides to discontinue its production or if the company goes out of business, researchers are left orphaned without means to repair or replace said device in the case of malfunction. If all the build plans are open, scientists can reproduce/repair it themselves or find other companies to produce them on demand. A concrete example [26] of this issue is provided by the European Organization for Nuclear Research (CERN) in which a specific hardware license [27] and several businesses protocols were created to ensure that hardware developed and sold to the project would have to comply with this license. This has enabled close collaborations in tool development between CERN and the hardware industry (as CERN employees could freely apply their expertise, both as end users and as engineers) and made the project robust to fluctuations in the market, since the necessary tools could be sourced from many suppliers.
4. In a FOSH-rich environment, information about material costs are easy to obtain and building plans are publicly available. Therefore, consumers are better equipped to decide whether the price being charged for a certain equipment is reasonable and to decide which route to take; they can invest time and effort in building, calibrating, and repairing their own tools (therefore saving money) or they can invest money to hire a company to do it for them (saving time and effort). In this new setting, saving potentials are quite large [28], without excluding companies from operating and offering valuable services for research and education.
5. The lower price tag on FOSH enables scientists in regions that are normally constrained by lack of funds to address scientific problems previously outside their reach. It fosters the discovery of untapped talent and paves the way for inside–out development, rather than relying on external aid and humanitarian assistance, a model that has had little success [29]. TReND in Africa [30], a volunteer-run nongovernmental organization, is leveraging this idea to train researchers in Africa on basic electronics and 3D printing as tools to develop labware and to involve academics in the global “maker movement” [31].
6. FOSH is also an excellent tool for education. As curious people learn how to build their own equipment, they are “forced” to learn about physics, electronics, and biology (see Table 1 for examples), creating new teaching possibilities for both schools and universities. Content can be taught in a “hands-on approach” by which students are challenged with a scientific question and try to solve it by thinking about what kind of experiments will be needed, building the instruments, gathering data, and drawing conclusions from them.

More examples of scientific equipment produced under the open source paradigm can be found for PCR machines, electrophysiology systems, supercomputers, prostheses, centrifuges, optics, spectrometers, diving robots, and even electron microscopes (Table 1 and Fig 1). The list is not exhaustive and new tools are added to online repositories and dedicated journals almost daily (Table 1).

**Fig 1:**
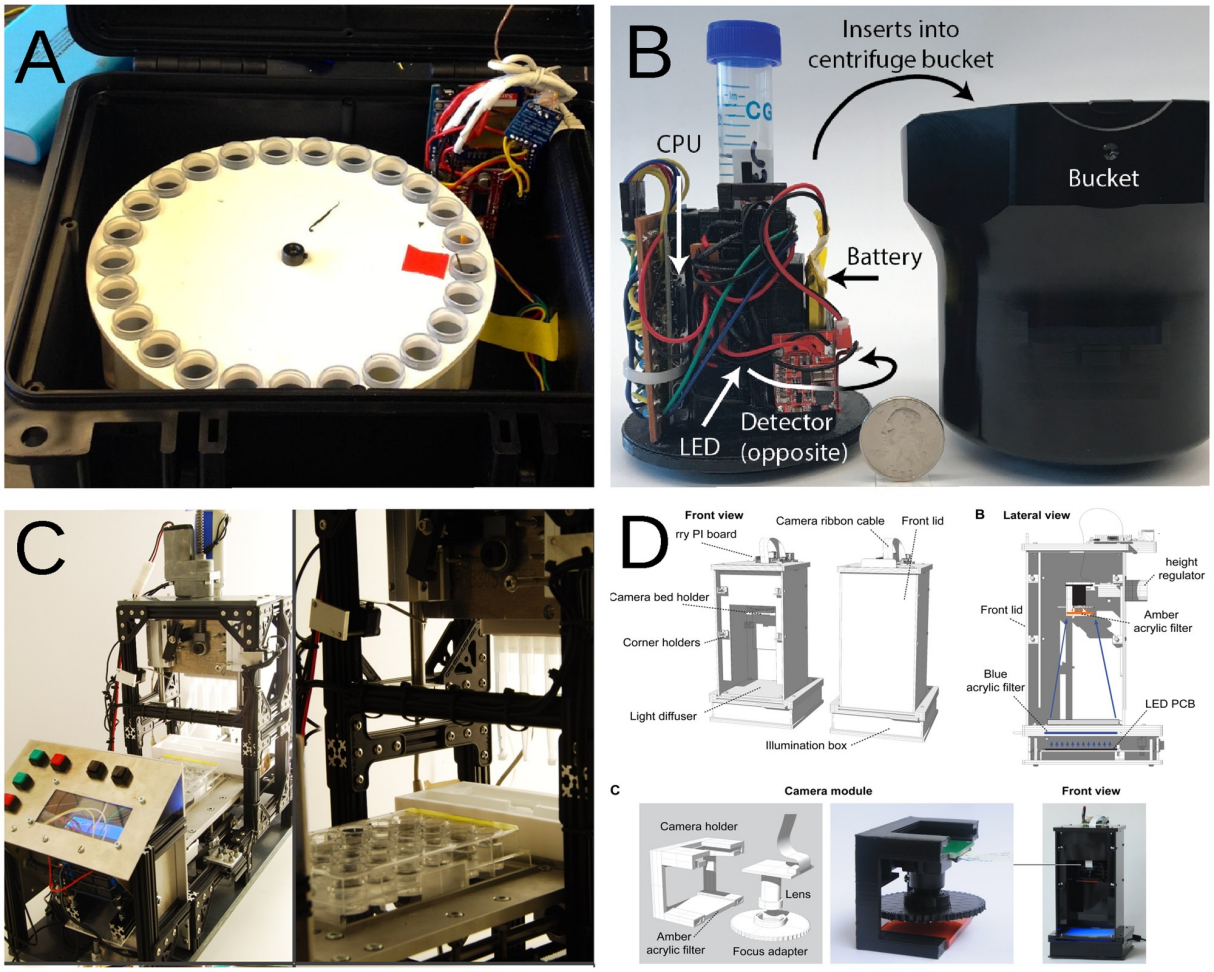
Open source hardware for research and education. A. Time-sorting pitfall trap and temperature logger [32]. B. “Smart” tube holder for real-time sample monitoring [33]. C. Pipetting robot for toxicological assays [34]. D. Low-cost multifluorescense system [35].

In this modern version of the “do-it-yourself” (DIY) tradition, people are developing tools with bits, bytes, resistors, and integrated circuits. They are still learning what works and what doesn’t, becoming ever more interdisciplinary, interconnected, and increasing the complexity of their designs. Taking advantage of the Internet, project repositories and information hubs are coming alive, breaking down the ivory tower, as scientists, makers, DIYers, and hobbyists interact on the same level, suggesting, contributing, and improving each others’ projects in a much richer peer review system, done by many, instead of just two or three pairs of eyes, and in an iterative manner. A prime example is the community growing around the Gathering for Open Scientific Hardware [36], set to have its third meeting from October 10th–13th 2018, bringing together ideas from different fields and creating collaborative, inclusive documentation, a manifesto [37], a roadmap ([38] for an ambitious but noble destination) to make open science hardware ubiquitous by 2025 and more importantly, a truly global community spirit [39], in which interdisciplinary and international events have been organized by the community members, among them the African Open Source Hardware Summit ([40], AfricaOSH), project Vuela! [41], and workshops on Open Source Laboratory equipment [42].

In 2013, Professor Marder was concerned by the number of times the phrase “the haves and the have nots” was being used in academia and the divide it represented. Even if the problem was solved by unlimited funding, it did not address the fact that, in the current system, institutions and labs that belong to the group that “has” are still in a system held hostage by their own tools. In order to solve this issue, we don’t necessarily need more money but rather need to reassess our relationship to knowledge and technology, how it determines our role in society, and how we want to spend grant money entrusted to us by the people. By making our tools and knowledge truly free, “haves and have nots” will not only erase the divide but will actually move together to a better way.

## Abbreviations

AfricaOSH: African Open Source Hardware Summit
CCD: charge-coupled device
CERN: European Organization for Nuclear Research
CPU: central processing unit
DIY: do-it-yourself
FOSH: free open source hardware
GDP: gross domestic product
